# O030. Treatment of orthostatic headache from spontaneous intracranial hypotension syndrome: single institutional experience of 326 cases

**DOI:** 10.1186/1129-2377-16-S1-A125

**Published:** 2015-09-28

**Authors:** Enrico Ferrante, Fabio Rubino, Ines Arpino, Federica Beretta, Alberto Citterio, Guglielmo Pero, Luca Quilici, Caroline Regna-Gladin, Mirko Maria Ferrante, Elio Agostoni

**Affiliations:** Neurosciences Department, Niguarda Cà Granda Hospital, Milan, Italy; Anaesthesiology Department, Niguarda Cà Granda Hospital, Milan, Italy; Anaesthesiology Department, Insubria University, Varese, Italy

## Background

Spontaneous intracranial hypotension (SIH) is characterized by orthostatic headache (OH), diffuse pachymeningeal enhancement on brain MRI and low CSF pressure. Treatment is usually conservative, but autologous epidural blood patch (EBP) has emerged as the most important non-surgical management.

## Materials and methods

From 1992 to 2015 we observed 326 patients (169 females and 157 males; age range 15-84; mean, 47 years) with OH from SIH according to the ICHD 2004 criteria. One hundred and sixteen performed a conservative treatment, while 210 underwent lumbar EBP with 15-50 ml (mean 28 ml) autologous blood. In 203 cases blood was mixed with contrast medium (1 ml of gadolinium [12 pts] and 5 ml of iopamidol [191 pts]), because about 30’ after EBP they underwent a spinal MRI or CT to document the blood spread into the epidural space. All patients were kept in a 30° Trendelenburg position for an hour before the procedure, during and for 24 h (52 pts) or 16 h (158 pts) after the procedure. Fifty-two patients were pre-medicated with acetazolamide (500 mg). The follow-up ranged from 6 months to 8 years.

## Results

OH disappeared after about 4-24 weeks in patients treated with conservative treatment and more quickly, in 16/24 hours, after EBP when the pts assumed an upright position. Twelve patients had a recurrence of SIH, 6 after a short period of time (1-4 week) and 6 after a long period of time (1-4 years). One pt had 3 relapses and another 2. Two patients did not recover after four EBP. Severe SIH complications were: cerebral venous sinus thrombosis: n. 4 pts (2 treated with EBP); coma (GCS: 5): 4 pts (3 treated with one EBP and 1 with three EBP); subdural hematoma: 48 pts (12 women, 36 men) with a thickness of the hematoma varying from 4 to 18 mm. Twenty pts performed hematoma evacuation (in 16 pts because of intracranial hypertension). EBP complications were in 90% of cases low back pain for 2-7 days, and in 5% of cases (10 patients) pneumocephalus, by use of air to locate the epidural space, as a result of accidental dural puncture or pressure gradient between the extra dural/subdural space, which resolved after a few days with symptomatic treatment.

## Discussion and conclusions

The lumbar EBP in Trendelenburg position appears to be safe and quickly effective in 99% of cases of OH from SIH, and in these, 94% after just a single treatment. While the conservative treatment seems to be effective in the longer period and sometimes with risk of severe complications.

Written informed consent to publication was obtained from the patient(s).
